# Vitexicarpin Acts as a Novel Angiogenesis Inhibitor and Its Target Network

**DOI:** 10.1155/2013/278405

**Published:** 2013-02-12

**Authors:** Bo Zhang, Lu Liu, Shiwen Zhao, Xu Wang, Liyang Liu, Shao Li

**Affiliations:** ^1^MOE Key Laboratory of Bioinformatics and Bioinformatics Division, TNLIST, Department of Automation, Tsinghua University, Beijing 100084, China; ^2^Joint Computational Center of Drug Discovery, Tianjin International Joint Academy of Biotechnology & Medicine, Tianjin 300457, China

## Abstract

Vitexicarpin (VIT) isolated from the fruits of *Vitex rotundifolia* has shown antitumor, anti-inflammatory, and immunoregulatory properties. This work is designed to evaluate the antiangiogenic effects of VIT and address the underlying action mechanism of VIT by a network pharmacology approach. The results validated that VIT can act as a novel angiogenesis inhibitor. Firstly, VIT can exert good antiangiogenic effects by inhibiting vascular-endothelial-growth-factor- (VEGF-) induced endothelial cell proliferation, migration, and capillary-like tube formation on matrigel in a dose-dependent manner. Secondly, VIT was also shown to have an antiangiogenic mechanism through inhibition of cell cycle progression and induction of apoptosis. Thirdly, VIT inhibited chorioallantoic membrane angiogenesis as well as tumor angiogenesis in an allograft mouse tumor model. We further addressed VIT's molecular mechanism of antiangiogenic actions using one of our network pharmacology methods named drugCIPHER. Then, we tested some key molecules in the VEGF pathway targeted by VIT and verified the inhibition effects of VIT on AKT and SRC phosphorylation. Taken together, this work not only identifies VIT as a novel potent angiogenesis inhibitor, but also demonstrates that network pharmacology methods can be an effective and promising approach to make discovery and understand the action mechanism of herbal ingredients.

## 1. Introduction

Antiangiogenic therapeutics proposed in 1971 has been widely used for the treatment of excessively angiogenic diseases such as cancer, psoriasis, and rheumatoid arthritis [1, 2]. In recent years, bioactive compounds of traditional Chinese medicine (TCM) herbs as a source of Antiangiogenic agents have played an important role in the discovery and development of anti-cancer drugs [3, 4]. For example, ginsenosides Rb1, Rb2, and Rg3 from *Panax ginseng* can inhibit tumor angiogenesis and metastasis by inhibiting the release of VEGF from tumour cells [5, 6]. Triptolide purified from *Tripterygium wilfordii* Hook F inhibits VEGF expression and secretion from endothelial cells (ECs) and decreases the expression of COX-1, COX-2, and 5-lipoxygenase [7, 8]. More recently, we and others found that sinomenine from *Sinomenium acutum* is a potent angiogenesis inhibitor and exerts synergistic inhibitory effects on ECs proliferation when combined with matrine from *Sophora flavescens* [9–11]. Thus, bioactive phytochemicals can serve as valuable lead compounds for developing derivatives, constituting a major source for discovering, and developing new antiangiogenic and anticancer drugs. Elucidation of the mechanisms of action of bioactive phytochemicals derived from TCM not only offers new insights into the action mechanism of herbs but also facilitates the ensuing use of bioactive phytochemicals as leads in drug development.

**Figure 1:**
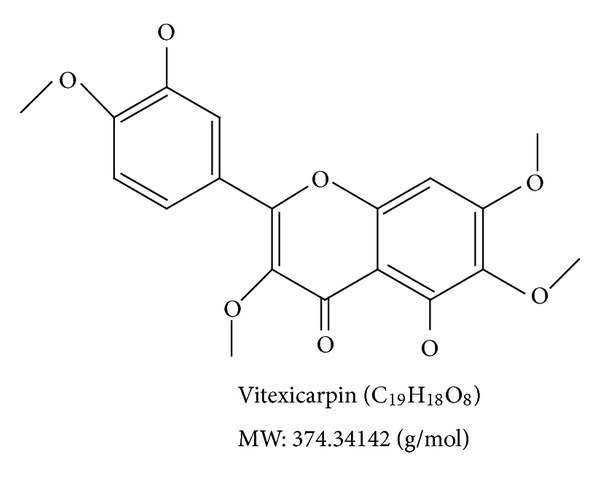
The chemical structure of vitexicarpin, a compound from *Vitex rotundifolia *(Man Jing Zi).

A variety of flavonoid substances, particularly those present in Chinese medicinal herbs, are hypothesized to be the potent kinase inhibitors and show a preventive effect on cancer [12]. Vitexicarpin is a flavonoid from the fruits of *Vitex rotundifolia *(Man Jing Zi), which is structurally typical flavones backbone (Figure 1). It has been documented that vitexicarpin exhibits broad cytotoxicity against human cancer cell lines [13, 14], exerts an inhibitory effect on T-lymphocyte proliferation [15], and prevents the TNF-*α*-induced vascular inflammation [16]. In addition to these activities, we firstly discovered that vitexicarpin inhibits the VEGF-induced ECs proliferation at a half-maximal inhibitory concentration (IC_50_) of 3.4 *μ*M [17]. It is speculated that vitexicarpin could inhibit tumor growth and inflammation responses by negatively regulating angiogenesis. However, the antiangiogenic mechanism of vitexicarpin remains elusive, and how to determine the promiscuous target-related proteins of vitexicarpin is also a challenge due to the less specific and low binding properties of small molecular compounds [18].

With the rapid development of network pharmacology [19] and systems pharmacology [20], recent works have demonstrated the value in using network approaches to provide a systematic insight into the molecular mechanisms of complex diseases (e.g., arrhythmias) [21], as well as therapeutic drugs (e.g., bafetinib) [22]. The information retrieved from biological network has been proven important for understanding the links between complex diseases and therapeutic drugs in a relatively unbiased and systematic manner [23, 24]. Therefore, network approaches leave room for *in silico* elucidation of drug action mechanisms, offering a new opportunity for drug discovery and development, especially for natural products and traditional Chinese medicine researches [25–28]. In our previous works, we proposed a novel theory of “Network Target” to analyze the mechanisms of action of herbal formula and its ingredients, and subsequently established a series of network-based methods as a starting point for TCM network pharmacology [11, 17, 23, 25, 26, 28, 29]. For example, we recently established a drugCIPHER method that can effectively predict target profiles for a drug or herbal compound [29]. The rationale of drugCIPHER is given a protein, if most of its neighbor proteins in the protein-protein interaction network are actually the known targets of FDA-approved drugs with similar chemical structures or therapeutic effects to a given compound, then this protein is most likely to be the target of this compound [29]. Thus, given a query drug or herbal compound, drugCIPHER can assign a score to each protein in the interaction network and describe the importance of the protein to the mechanism of actions of this drug or herbal compound.

Here, we tried to give an example of the application of TCM network pharmacology on the research of herbal compounds and took a “top-down” approach to identify the Antiangiogenic target network of vitexicarpin. Taking advantage of the *in vitro* and *in vivo* models of angiogenesis, we systematically examined the effects of vitexicarpin on different steps and players involved in angiogenesis. We then used drugCIPHER to predict the target profiles of vitexicarpin and found that pathways enriched in target profiles can explain the therapeutic activities of vitexicarpin. We also addressed the molecular mechanisms underlying vitexicarpin's Antiangiogenic effect by identifying the possible target network involved in VEGF pathway. Eventually, experimental validation of the target network revealed that reduced phosphorylation states of SRC and AKT are in response to the anti-angiogenesis actions of vitexicarpin. 

## 2. Materials and Methods

### 2.1. Cell Lines, Cell Culture, and Reagents

Primary human umbilical vascular endothelial cells (HUVECs) were purchased from ScienCell Research Laboratories. HUVECs were grown onto gelatin-coated 10 cm^2^ culture dishes in a standard endothelial cell medium (ECM) supplemented with 5% fetal bovine serum (FBS; Hyclone Laboratories), 50 *μ*g/mL endothelial cell growth supplement (ECGS; Sigma), 50 IU/L penicillin, and 50 mg/L streptomycin at 37°C in a humidified 5% CO_2_/95% air incubator. Vitexicarpin was purchased from National Institute for the Control of Pharmaceutical and Biological Products, Beijing, China. A 50 mM solution of vitexicarpin was prepared in DMSO, stored at −20°C and protected from light, and then diluted as needed concentrations in culture medium.

### 2.2. *In Vitro* Angiogenesis Assay 

#### 2.2.1. Migration Analysis by Wound Healing and Transwell Assay

 HUVECs migration was evaluated by both the CytoSelect 24-well Wound Healing Assay and Transwell Migration Assay. A CytoSelect Wound Healing Assay kit (CBA-120-5) purchased from Cell Biolabs (San Diego) was used for this assay. HUVECs suspended in culture medium containing 5% FBS were seeded at 10,000 cells per well on 24-well culture dishes precoated with 0.1% gelatin (Sigma), and then put the CytoSelectTM Wound Healing Insert into the plate wells. After overnight incubation, the inserts were removed from the well to generate a 0.9 mm “Wound field.” The cells were incubated with 20 *μ*g/mL mitomycin C for 1 h to deactivate HUVEC proliferation. After that, the cells were washed with PBS to remove debris and incubated with or without VEGF and different concentrations of vitexicarpin. After 12 h of incubation, images were taken by a phase contrast microscope (Olympus) and the wound size was determined [30]. 

The chemotactic motility of HUVECs was assayed using Transwell chamber with 6.5 mm diameter noncoated filter membrane (pore size, 8 *μ*m; Corning, Inc.). Endothelial cell medium with 0.1% FBS supplemented with 10 ng/mL VEGF was used as chemoattractant in the bottom chamber, and 5 × 10^4^ HUVECs pretreated with mitomycin C in 200 *μ*L ECM (0.1% FBS) with different concentrations of vitexicarpin were seeded in the top chamber. The cells were migrated for 4 h and the nonmigrated cells on the top surface of the membrane were removed by wiping with a cotton swab. The migrated cells on the lower side of the membrane were fixed with cold 4% paraformaldehyde for 1 h and then stained with hematoxylin and eosin (H&E). Chemotaxis was quantified by manually counting the migrated cells with inverted microscope at ×200 magnification. Four independent areas were counted for each assay.

#### 2.2.2. Apoptosis Analysis by Annexin V/Propidium Iodide (PI), Hoechst 33342, and DNA Ladder

Vitexicarpin-induced apoptotic death of HUVECs was detected by Annexin V and PI staining and flow cytometry. After vitexicarpin treatment, floating and adherent cells were collected and washed with PBS twice, and stained by using Vybrant Apoptosis Assay Kit (Invitrogen) following the standard protocol provided by the manufacturer. After staining, apoptotic cells were quantified by flow cytometry and examined using Olympus fluorescence microscope. The rates of early-stage apoptosis cells were demonstrated to be Annexin V positive and PI negative. Vitexicarpin-induced DNA condensation of apoptotic cells was assessed by Hoechst 33342 staining. HUVECs were starved with 0.1% FBS culture medium and then treated with or without VEGF (10 ng/mL) and different concentrations of vitexicarpin. After incubation for another 12 h, both floating and adherent cells were pooled and stained with Hoechst 33342 (Molecular Probes, Eugene, OR). The nuclei of apoptotic cells with characteristic nuclear fragmentation were counted in randomly chosen fields using an Olympus fluorescence microscope and expressed as a percentage of the total cell number. For assays of DNA ladder, cells were collected and lysed. RNase was added to the lysate and incubated for 30 minutes at 37°C. Proteinase K and SDS were added, followed by incubation at 50°C for 16 hours. DNA was extracted, precipitated, and electrophoresed. The stained gel was visualized by ultraviolet (UV) light and photographed. 

### 2.3. *Ex* and *In Vivo* Angiogenesis Assay

#### 2.3.1. Tube Formation Assay

A matrigel tube formation assay was performed to assess *ex vivo* angiogenesis. Growth factor-reduced Matrigel was thawed at 4°C and placed in prechilled 24-well culture plates (200 *μ*L/well) and set at 37°C for 30 min. The tube formation assay was performed employing HUVECs within six passages. A suspension of 5 × 10^4^ HUVECs in 500 *μ*L ECM (supplemented with 0.1% FBS and 10 ng/mL or 50 ng/mL VEGF) were seeded in duplicate into Matrigel-precoated 24-well plates in the presence of different concentrations of vitexicarpin. After 16 h of interventions, the cultures were photographed using ×40 Olympus inverted microscope. The length of the tube was calculated by importing digital images into NIH ImageJ. Inhibition percentage was expressed using solvent control as 100%.

#### 2.3.2. CAM Assay

As described by Richardson and Singh [31], fertilized White Leghorn chicken eggs (8 per group) were incubated at 37°C at constant humidity. On day 3, a square window was opened in the shell after removal of 2-3 mL of albumen in order to detach the developing CAM from the shell. The window was sealed with a glass, and the eggs were returned to the incubator. On day 8, growing CAMs were treated as follows: 1 mm^3^ sterilized gelatin sponges were implanted on top of the growing CAMs and adsorbed with 2 *μ*L PBS with 0.1% DMSO as vehicle control, 2 *μ*L PBS containing 50 ng VEGF as angiogenic model control, 2 *μ*L PBS admixed to VEGF and VIT at 0.1 *μ*M to 5 *μ*M. At day 12, CAMs fixed with Bouin's fluid were photographed with a stereomicroscope equipped with a CCD camera and image analyser system (Olympus). The angiogenic response was assessed as the number of vessels converging toward the sponges. 

#### 2.3.3. Allograft Model

Murine sarcoma S180 cells (1 × 10^7^ cells/body) were suspended in a mixture of DPBS and Matrigel (BD BioSciences, San Jose, CA, USA) and subcutaneously implanted into the axilla of female Kunming mice (3-month old, 15–25 g) from Experimental Animal Center of the Academy of Military Medical Sciences (Beijing, China) on day 0. Tumor-bearing animals were randomized into vehicle or vitexicarpin treatment groups (10 mice per group) on day 1. Mice were injected intraperitoneally with vehicle (Normal saline, NS) or dilutions of vitexicarpin (100, 150 mg/kg body weight, dissolved 70% DMSO and 30% NS) on every other day for 2 weeks. Mice were monitored daily and tumor volumes were measured twice weekly. Tumors were harvested after two weeks and either formalin-fixed and paraffin-embedded and snap-frozen for H&E staining and immunohistochemical analysis.

### 2.4. Western Blot and Gelatin Zymography

 Cell lysates were prepared in 100 *μ*L of denaturing lysis buffer (2% SDS, 50 mM Tris, 2 mM EDTA). The protein extracts were subjected to SDS-PAGE and transferred to a nitrocellulose membrane (Pall Corp.). After the transfer, the membranes were incubated with blocking solution and probed with various antibodies followed by washing. Protein levels were assessed by immunoblotting with the specific antibodies and detected by the chemiluminescence detection system. Anti-AKT1, anti-phospho-AKT1, anti-SRC, anti-phospho-SRC, anti-procaspase3, anti-procaspase7, anti-PARP, anti-cleaved-PARP, and anti-*β*-actin antibodies were purchased from Cell Signaling Technology. 

Gelatin zymography was used to examine the activity of MMP2. All media were collected and subjected to SDS-PAGE using 0.01% w/v gelatin containing 10% polyacrylamide gel. After electrophoresis, the gels were equilibrated in 50 mM Tris-HCl (pH 7.5) with 2.5% Triton X-100 for 30 min at room temperature. Gels were then incubated at 37°C for 20 h in an incubation buffer [50 mM Tris-HCl (pH 7.5), 150 mM NaCl, and 10 mM CaCl_2_, 1 mM ZnCl_2_ and 0.02% NaN_3_], and then stained with 0.25% Coomassie Blue R250.

### 2.5. The Target Network of Vitexicarpin and Functional Analysis

The target-related proteins of vitexicarpin were predicted using the drugCIPHER-CS step in our drugCIPHER method, whose good performance on target prediction was demonstrated in our previous work [29]. Briefly, based on the “Like attracts like” rationale, the drugCIPHER method predicts the target profile for a given compound mainly by correlating the drug similarity network around this compound and genome-wide proteins in the protein-protein interaction network. The drugCIPHER score refers to the likelihood of compound-target interaction calculated from the correlation between the query compound's structure similarity vector in the drug space and the target-related gene's functional similarity vector in the target space. The resulting target proteins with high likelihoods are considered as the potential drug targets. In this study, we obtain the known drug-target interactions from DrugBank (version: January, 2012) [32]. The chemical structure relation is calculated based on Tanimoto coefficient [33]. The human protein interaction network is constructed from HPRD [34], BIND [35], IntAct [36], MINT [37], and OPHID [38]. The assumption is that drugs with similar target profiles predicted by drugCIPHER may target a common network. In order to construct the target network of VIT when inhibiting VEGF-induced angiogenesis, we selected FDA-approved drugs whose target proteins can fall into the VEGF pathway defined by KEGG. Then, we used the Pearson's linear correlation coefficient between the predicted target profiles of each selected drug and vitexicarpin as a measure of drugCIPHER-based compound-drug similarity. It is calculated by the following equations

(1) $$\begin{array}{c} r=\frac{N\left(\sum XY\right)-\left(\sum X\right)\left(\sum Y\right)}{\sqrt{\left[N\sum X^{2}-{\left(\sum X\right)}^{2}\right]\left[N\sum Y^{2}-{\left(\sum Y\right)}^{2}\right]}}, \end{array}$$

where *X*, *Y*denote the drugCIPHER-CS score of vitexicarpin and FDA-approved drugs, respectively, and *N* is the total number of proteins in the target profiles. 

For functional analysis, we use DAVID web server to analyze the enriched KEGG pathways for the top 10% target-related proteins of vitexicarpin with a false discovery rate less than 0.05 by the Fisher Exact test [39]. We only selected the KEGG pathways with *P* value less than 0.05 after Bonferroni's correction.

### 2.6. Docking Analysis

The high-resolution protein structures of SRC and AKT1 were obtained from the refined X-ray crystal structure of 2H8H.pdb and 3MVH.pdb, which is available from the Protein Data Bank. The Autodock 4.0 suite of programs uses an automated docking approach that allows ligand flexibility as described to a full extent elsewhere [40]. Default parameters were used as described in the AutoDock manual. The protein structure was prepared using the PyMol and AutoDockTools and used for flexible docking studies with AutoDock 4.0 after extraction of the crystal ligand. 

### 2.7. Statistical Analysis

The data are presented as mean ± SD, and statistical comparisons between groups were performed using Student's *t*-test. *P* < 0.05 was considered statistically significant.

## 3. Results

### 3.1. Vitexicarpin Inhibits VEGF-Induced Lateral, Longitudinal Migration and Tube Formation of Endothelial Cells

**Figure 2:**
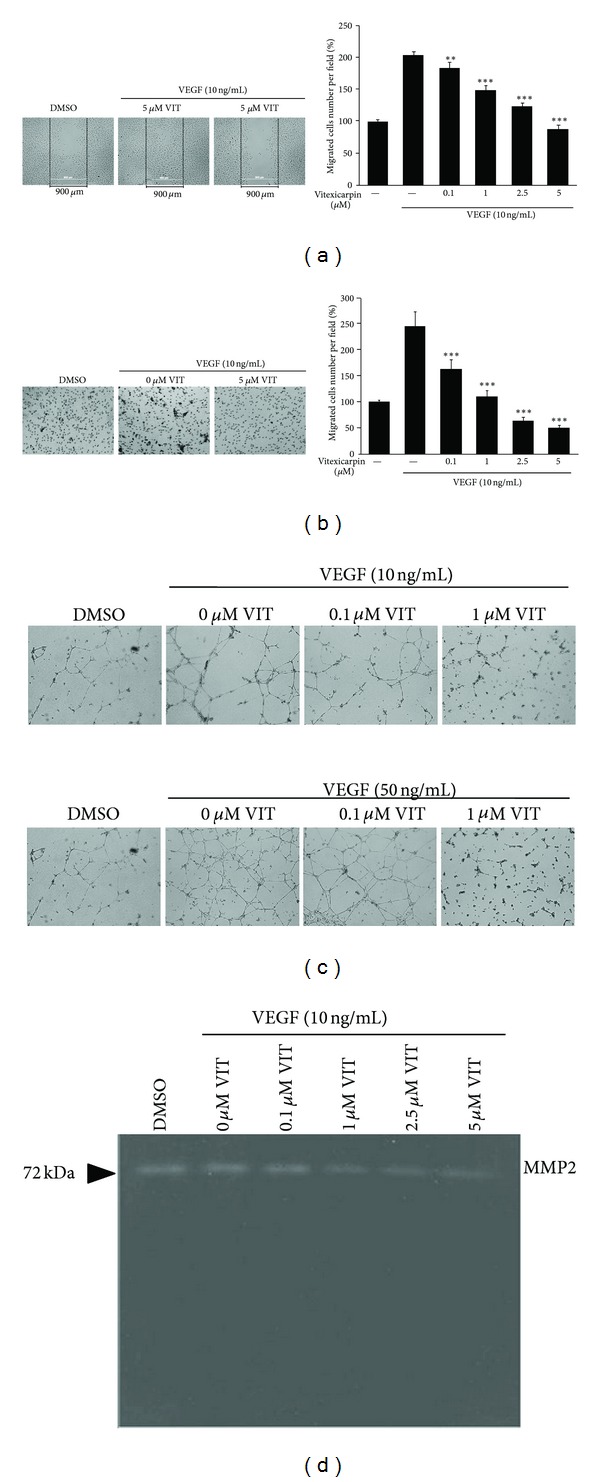
Vitexicarpin inhibits VEGF-induce migration and tube formation. Vitexicarpin at a range of concentrations (0.1 *μ*M–5 *μ*M) concentration dependently inhibits HUVEC migration caused by wound healing (a) and HUVEC invasion using transwell chamber chemotaxis assay (b). (c) Vitexicarpin inhibits VEGF-induced tube formation of HUVECs. Representative results of HUVECs on Matrigel in the absence or presence of 10 ng/mL or 50 ng/mL VEGF plus different concentrations of vitexicarpin. (d) Vitexicarpin inhibits the enzyme activity of MMP2. Gel Zymography analysis of the effect of vitexicarpin on MMP2 activity. **P < 0.01; ***P < 0.001 compared with VEGF group. Error bars represent ± SD of experiments.

To evaluate the effect of vitexicarpin on VEGF-stimulated endothelial cell migration, wound healing and transwell cell migration assays were performed to explore whether vitexicarpin could inhibit the VEGF-induced motility of HUVECs. As shown in Figure 2(a), vitexicarpin inhibits VEGF-induced HUVECs migration in a concentration-dependent manner, with half-maximal inhibition at ~2.5 *μ*M. Vitexicarpin significantly inhibited VEGF-induced lateral migration and decreased wound healing potential at 5 *μ*M (Figure 2(a)). Next, the effects of vitexicarpin on the longitudinal migration of endothelial cells were investigated using transwell assays. The number of HUVECs that migrated in the Transwell assay was higher in the presence of VEGF than in the control. At 0.1 *μ*M, vitexicarpin reduced the longitudinal EC migration induced by VEGF (Figure 2(b)). These results suggested that vitexicarpin inhibited cell migration by inhibiting the SRC pathway downstream protein kinase activities.

To examine the potential effects of vitexicarpin on the capillary structure formation of endothelial cells, we investigated how vitexicarpin affects VEGF-induced tube formation using a Matrigel assay. When HUVECs are seeded on Matrigel, they should form elongated and robust capillary-like structures. This phenomenon can be facilitated by the proangiogenic factor VEGF, whereas vitexicarpin effectively blocked the VEGF-induced tube formation after 16 h of incubation (Figure 2(c)). 0.1 *μ*M vitexicarpin partly inhibited endothelial tube formation on Matrigel and 1 *μ*M vitexicarpin completely blocked the formation of the tubular structures. To further understand the inhibitory effects of vitexicarpin on tube formation, we also examined the effect of vitexicarpin on the activity of MMP2 which results in the extracellular matrix degradation and enhances tube formation. As shown in Figure 2(d), we found that vitexicapin inhibited the activity of MMP2 and thus caused an inhibitory action of tube formation. These results demonstrated that vitexicarpin can block VEGF-induced *in vitro* and *ex vivo* angiogenesis by inhibiting lateral, longitudinal migration and tube formation of endothelial cells.

### 3.2. Vitexicarpin Inhibits VEGF-Induced HUVECs Proliferation and Induces Cell Cycle Arrest

**Figure 3:**
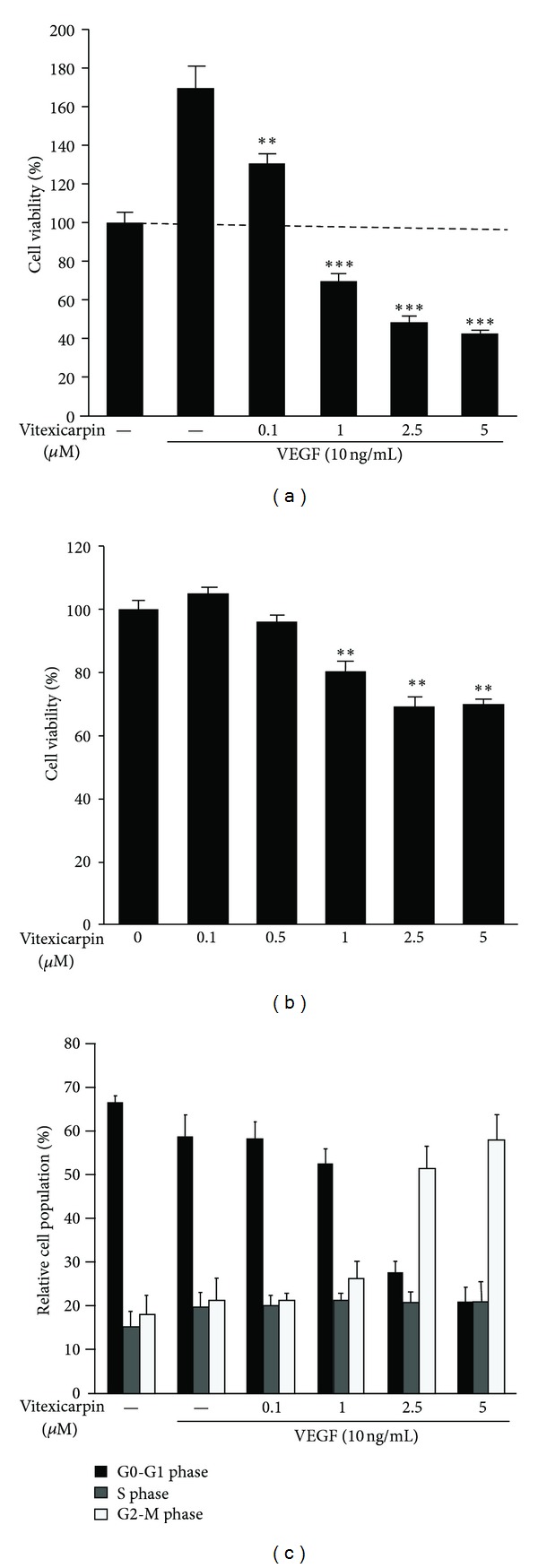
Vitexicarpin inhibits VEGF-induced proliferation of endothelial cells. (a) Vitexicarpin inhibits VEGF-induced endothelial cells viability in a dose-dependent manner. Cell viability was quantified by CCK-8 assay. (b) Effects of vitexicarpin at indicated concentrations on endothelial cells viability under normal culture conditions. (c) Cell cycle analysis showing G2/M arrest in cells treated with vitexicarpin at indicated concentrations. **P < 0.01; ***P < 0.001 compared with VEGF control.

To evaluate the antiproliferative effect of vitexicarpin on VEGF-stimulated endothelial cells, we examined the inhibitory effects of vitexicarpin on the viability of HUVECs stimulated with VEGF165 by using CCK-8. HUVECs (2 × 10^4^ cells/well) were incubated for 24 h in 96-well microplates with various final concentrations of vitexicarpin (0, 0.1, 1, 2.5, and 5 *μ*M). As shown in Figure 3(a), vitexicarpin can significantly inhibit VEGF-mediated HUVECs viability at concentrations as low as 1 *μ*M, with an IC_50_ of approximately 2.5 *μ*M. As a solvent control, we also examined the effect of 0.1% DMSO on cell viability. Upon supplementation of the culture medium with 0.1% DMSO, our data indicated that 0.1% DMSO has no effects on HUVEC proliferation and viability. The cytotoxic activity of vitexicarpin against HUVECs was assessed without the supplementation of proangiogenic factors in 5% FBS-containing endothelial cell medium (condition did not support proliferation). Vitexicarpin treatment at 5 *μ*M for 24 h only decreased the percentage of metabolically viable HUVECs by approximately 25% (Figure 3(b)). To assess whether vitexicarpin regulated cell cycle progression, we additionally performed PI analysis by FACS. After HUVECs were treated with 10 ng/mL VEGF in the presence or absence of various concentrations of vitexicarpin for 24 h, the percentage of cells in G0/G1, S, and G2-M phases was monitored. Vitexicarpin reduced the percentage of G0–G1 phase cell from 59.24 to 19.81%, and a concomitant accumulation of cells in G2-M phase from 20.98 to 59.55% was observed. VEGF induced HUVECs to enter S phase, whereas the addition of vitexicarpin did not reduce S phase entry (Figure 3(c)). These results suggest that vitexicarpin could arrest the cell cycle progression.

### 3.3. Vitexicarpin Induced the Apoptotic Death of HUVECs

**Figure 4:**
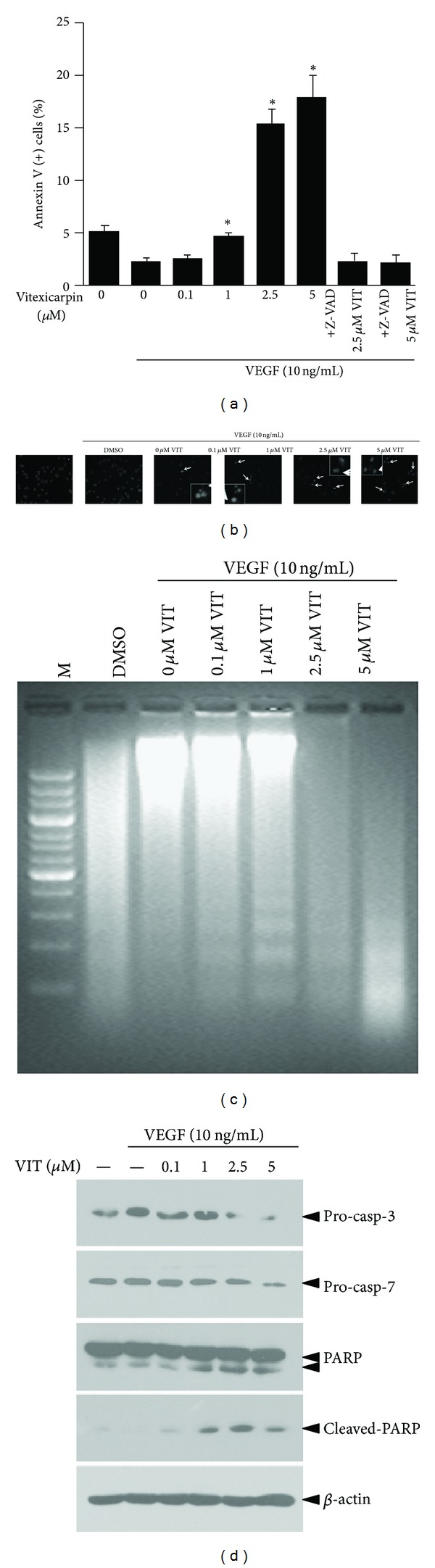
Vitexicarpin induces apoptosis of VEGF-stimulated endothelial cells. (a) Vitexicarpin increases annexin V (+) cells at indicted concentrations. (b) Hoechst 33342 staining of apoptotic endothelial cells. Cells are magnified in inset figure. (c) DNA fragment in endothelial cells treated with vitexicarpin at indicted concentrations for 12 h. (d) Effects of vitexicarpin on pro-casp-3, pro-casp-7, PARP, and cleaved-PARP. *β*-actin is used as a loading control. **P < 0.01; ***P < 0.001 compared with VEGF group.

To verify the proapoptotic effects of vitexicarpin on VEGF-stimulated HUVECs, we evaluated the apoptotic cell death effect of vitexicarpin by annexin V-PI staining and flow cytometry, in which only annexin V-stained cells were considered as early apoptotic cells (Figure 4(a)). We also visualized the apoptotic effects of vitexicarpin by using Hoechst 33342 staining. HUVECs were treated with a range of concentrations of the vitexicarpin for 24 h. Examination of the HUVECs upon vitexicarpin treatment revealed that the appearance of morphologic characteristics of apoptosis, such as plasma membrane disintegration, chromatin condensation, and nuclear fragmentation, was significantly induced by 1 *μ*M vitexicarpin, as indicated in Figure 4(b). These results suggest that vitexicarpin induces early-stage apoptosis in HUVECs in the presence of VEGF. DNA electrophoresis using samples isolated from vitexicarpin (5 *μ*M)-treated HUVECs confirmed DNA fragmentation (Figure 4(c)). To gain more insight into how vitexicarpin induces apoptosis in endothelial cells, we pretreated cells with the pan-caspase inhibitor Z-VAD-fmk. This inhibitor reduced the percentage of annexin V-positive cells among vitexicarpin-induced apoptotic HUVECs, indicating that the classical caspase pathway was required for the apoptosis induced by vitexicarpin (Figure 4(a)). The potential mechanisms of vitexicarpin-mediated apoptosis were explored by Western blot analysis of some of the proteins involved in the apoptotic process (Figure 4(d)). A clear decrease in pro-caspase-3 and pro-caspase-7 expression was observed using 2.5 *μ*M and 5 *μ*M vitexicarpin, respectively. We also observed a concentration-dependent increase in the expression of the cleaved-poly (ADP-ribose) polymerase (PARP). These results demonstrated that vitexicarpin inhibited proliferation and induced early and late apoptosis in VEGF-induced HUVECs.

### 3.4. Vitexicarpin Inhibits VEGF-Induced Angiogenesis *Ex* and *In Vivo*

**Figure 5:**
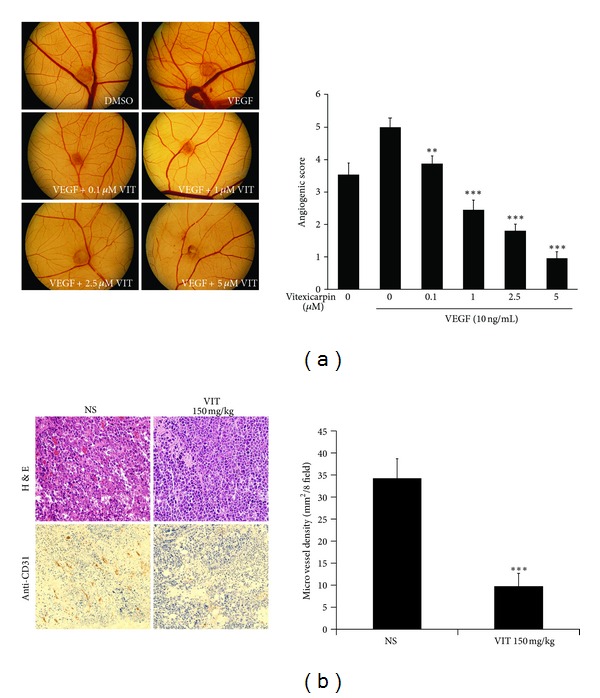
Vitexicarpin inhibits VEGF-induced angiogenesis *ex vivo* and *in vivo*. (a) Vitexicarpin inhibits VEGF-induced angiogenesis in the CAM assay *in vivo*. In comparison to DMSO group, the application of VEGF (10 ng/mL) induced a strong vascularization. In contrast, the simultaneous application of VEGF and vitexicarpin blocked VEGF-induced angiogenesis. **P < 0.01; ***P < 0.001 compared with VEGF group; bars, ± SD. (b) H&E staining (upper panels) and anti-CD31 stained blood vessels (lower panels) of tumor sections from NS and vitexicarpin (150 mg/kg). Vitexicarpin inhibits tumor angiogenesis in allograft mouse tumor model. All images, ×200 magnification.^  ∗∗∗^ *P* < 0.001 compared with NS group; Error bars, ± SD.

To assess the effect of vitexicarpin on angiogenesis *in vivo*, blood vessel growth was stimulated on the chorioallantoic membranes (CAMs) with VEGF in the presence or absence of different concentrations of vitexicarpin (0.1–5 *μ*M). This assay measures developmental angiogenesis, and it is routinely used to obtain the first indication of angiogenic activity *in vivo*. VEGF induced a robust angiogenic response, whereas vitexicarpin disrupted VEGF-mediated angiogenesis (Figure 5(a), right panel). Quantitative analysis revealed that VEGF caused a 3.2-fold increase in the number of newly formed blood vessels compared with that of medium alone (Figure 5(a), left panel; *P* < 0.001). The development of vasculature at day 10 treated with 0.1 *μ*M, 1 *μ*M, 2.5 *μ*M, and 5 *μ*M vitexicarpin caused a 24, 36, 45, and 84% reduction in the infiltration of blood vessels, respectively. These results suggested that vitexicarpin is a potent antiangiogenic molecule *ex vivo*. 

To examine the inhibitory effect of vitexicarpin on tumor angiogenesis and growth, we used an allograft mouse tumor model. The primary tumors (after 14 days) were dissected, fixed, and imbedded. Tumors from the mice with vitexicarpin treatment were smaller than that from NS group, suggesting that vitexicarpin inhibits tumor growth *in vivo*. H&E staining showed that treatment of tumors with vitexicarpin appeared to increase the extent of necrosis within tumor (Figure 5(b)). Furthermore, there were profound differences in the number of blood vessels in the tumor tissue from NS and vitexicarpin group (Figure 5(b)). Based on anti-CD31 staining, microvessel density (MVD) in tumor tissue from NS group was 34.4 ± 4.4 mm^2^/field, whereas that in vitexicaprin-treated group was 10 ± 2.7 mm^2^/field (*P*  value = 6.14*E* − 06; Figure 5(b)), indicating that vitexicarpin significantly inhibited tumor angiogenesis.

### 3.5. Predicted Target Profiles Reveal the Antitumor and Antiangiogenic Activities of Vitexicarpin

To identify the target-related proteins of vitexicarpin, we used drugCIPHER-CS [29] to calculate its likelihood to bind to all the 13,388 proteins in the interaction network. Here we only assembled and ranked the 2,039 druggable proteins which are often known targets in the DrugBank. 204 target proteins (top 10%) represented the potential targets of vitexicarpin. 

Enrichment analysis helps to better understand the biological functions of target proteins and pathways involved in the activities of vitexicarpin. By establishing a set of the known angiogenesis-related drug targets (i.e., the known targets of angiogenic drugs in DrugBank), we found that top 10% target proteins are significantly enriched in the set of known angiogenesis-related drug targets (*P* = 3.63*E* − 05). By using top 10% targets of vitexicarpin, we also found significantly enriched KEGG pathways vitexicarpin may affect cancer-related pathways (e.g., Non-small cell lung cancer, Prostate cancer, Glioma, Pathways in cancer, etc.), T cell receptor signaling pathway, and VEGF signaling pathway. Interestingly, it has been previously reported that vitexicarpin exhibits broad cytotoxicity against human cancer cell lines and exerts an inhibitory effect on T-lymphocyte proliferation. The comparison results with literature suggest that target profiles predicted by drugCIPHER can reveal the polypharmacological activities of vitexicarpin.

### 3.6. Target Network Prediction and Validation of Vitexicarpin When Inhibiting Angiogenesis

**Figure 6:**
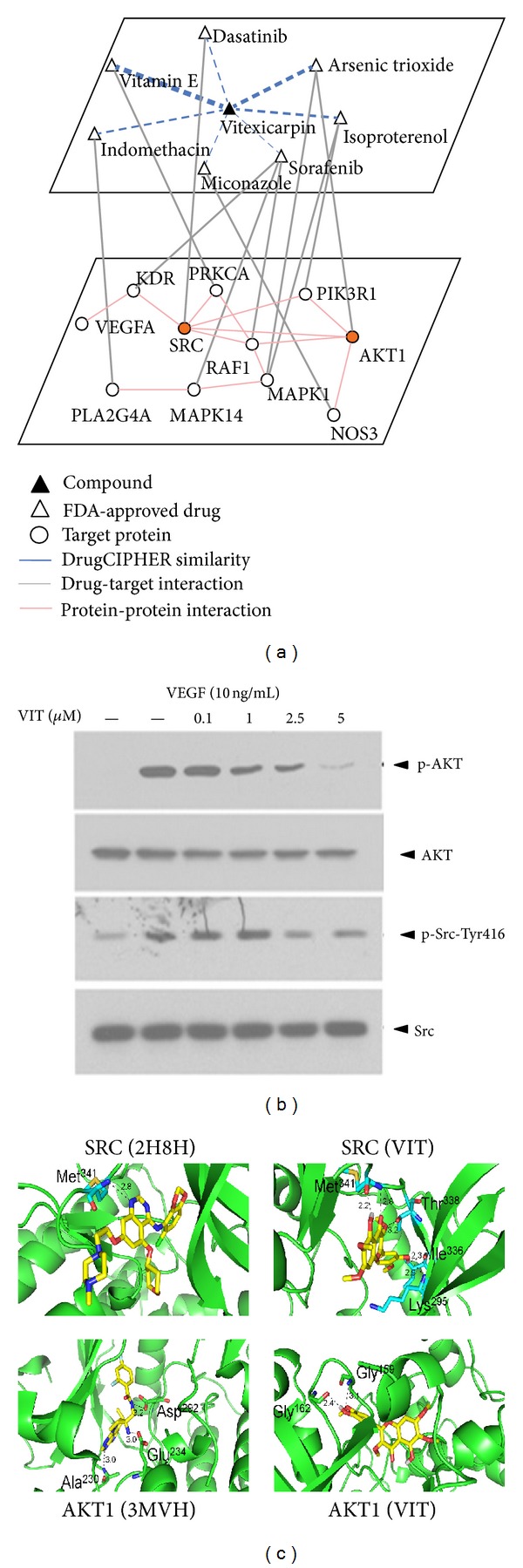
The constructed target angiogenesis-related network of vitexicarpin and experimental validation of two key molecules. (a) Network diagram showing vitexicarpin targeting network when inhibiting angiogenesis. The dot line thickness denoted by drugCIPHER similarities is the drugCIPHER scores' correlation coefficients between drugs and vitexicarpin. (b) Western blot analysis was performed to examine the changes of phosphoprotein levels of p-SRC and p-AKT in the vitexicarpin-treated HUVECs. Proteins were extracted from the cultured HUVEC at 30 min after vitexicarpin treatment and probed with proper dilutions of specific antibodies. Vitexicarpin inhibits VEGF-induced p-SRC and p-AKT phosphorylation in a dose-dependent manner. (c) Comparison of binding interactions of quinazoline inhibitor (PDB 2H8H) and vitexicarpin with SRC kinase (upper panel) and comparison of binding interactions of WFE (PDB 3MVH) and vitexicarpin with AKT1 (lower panel). Vitexicarpin is in stick. Carbon atom for vitexicarpin (yellow). Hydrogen bonds are displayed (dark dashed lines). The value on the dashed lines denotes the distance (Å) of H-bonds.

Having shown that the target profiles predicted by drugCIPHER can help improve the capacity to comprehensively understand the mechanisms of herbal compounds, we further set out to elucidate the Antiangiogenic mechanisms of vitexicarpin by network pharmacology approach. According to the principle of drugCIPHER, we identified the possible target network by which vitexicarpin can exert inhibition effects on VEGF-induced angiogenesis. First, we collected 58 FDA-approved drugs directly targeting 18 proteins which can fall into the VEGF pathway. Among the 18 proteins, 11 of them can link together into one network through direct protein physical interaction at the protein interaction level (Figure 6(a)). Then, we calculated the correlations between 58 FDA-approved drugs' and vitexicarpin's profiles. Direct target proteins of FDA-approved drugs that tend to be more similar to vitexicarpin in profile clustering are the potential targets of vitexicarpin. We therefore reasoned that a specific network that can connect all the direct target proteins of drugs with profiles highly similar to vitexicarpin could be used to estimate its possible targeting functional network, for example, VEGF pathway. Using this principle, we identified the target network composed of 11 proteins, suggesting that vitexicarpin can achieve the Antiangiogenic effects through targeting key molecules in this network target embedded in the VEGF pathway (Figure 6(a)).

To preliminarily assess the predicted targets of vitexicarpin, we selected two key kinase molecules in VEGF-induced ECs migration and proliferation pathways, SRC and AKT, whose drugCIPHER scores are ranked top 2 in this network target. The experimental results demonstrated that VEGF significantly increased SRC kinase phosphorylation at Tyr416, but this increase was blocked by vitexicarpin in a concentration-dependent manner (Figure 6(b)). Half-maximal effects were obtained at a concentration of ~2.5 *μ*M. Consistently, AKT1, which is a key signal mediator in VEGF-stimulated HUVECs, was phosphorylated by VEGF, and AKT1 activation was suppressed by vitexicarpin in a concentration-dependent manner (Figure 6(b)). The result showed that vitexicarpin significantly inhibited VEGF-induced cell migration and proliferation and induced apoptosis through inhibition of the SRC and AKT pathway downstream molecules. 

To evaluate the possibility of SRC and AKT1 as the direct targets of vitexicarpin, we performed docking analysis to examine the mode of vitexicarpin-binding SRC and AKT1. The available X-ray crystal structures of kinases from the PDB database were used for validation of target prediction results. We found that the binding energy of vitexicarpin with SRC and AKT1 is −9.23 kcal/mol and −9.21 kcal/mol, respectively. Vitexicarpin was docked into the ligand-binding sites and occupied the hydrophobic pocket of SRC and AKT1 (Figure 6(c)). Compared to published complex of SRC (2H8H), vitexicarpin was able to maintain five hydrogen bonds with Met^341^, Lys^295^, Ile^336^, and Thr^338^ at the active site of SRC. In addition, the vitexicarpin/AKT1 model generated two hydrogen bonds with Gly^159^ and Gly^162^ and two *π*-*π* interactions compared with AKT1 complex (3MVH). These results indicate the potential of SRC and AKT1 as direct targets of vitexicarpin, which deserve further experimental verifications.

## 4. Discussion

In recent years, substantial effort has been dedicated to identifying Antiangiogenic agents from TCM herbs [4, 41]. Vitexicarpin is a bioactive flavonoid from *V. rotundifolia*, which can inhibit tumor growth and inflammation. However, little information is known about its functions in angiogenesis. In this study, we demonstrate that vitexicarpin (0.1–5 *μ*M) exhibited Antiangiogenic activities *in vitro*, as shown in the results of endothelial cells migration, proliferation, and Matrigel tube formation assays (Figures 2 and 3). Further studies using flow cytometric analysis, DNA fragment, and caspase-3 blotting indicated that vitexicarpin (0.1–5 *μ*M) inhibited ECs proliferation via cell cycle arrest and induction of apoptosis (Figure 4). Consistent with our *in vitro* results, 5 *μ*M vitexicarpin inhibited the angiogenesis sprouting from CAM (Figure 5(a)). In addition, vitexicarpin impaired vascularization in allograft mouse tumor model (Figure 5(b)). It should be noted that therapeutic effects of Man Jing Zi as an antiarthritis herb, described in Ri-Hua-Zi-Ben-Cao, are deciphered by the Antiangiogenic activity of vitexicarpin.

At present, one of the major challenges in the use of herbal compounds in drug discovery is the unexpected interactions with on-target and off-target proteins due to the less specific binding characteristics of small molecules [18, 42]. In addition, herbal compounds have more complex stereochemical and physicochemical properties compared to synthetic small molecules [18, 43]. Thus, exploiting the promiscuous targets of herbal compounds enables a deeper understanding of the global perturbation of these compounds induced at the molecular level [44, 45]. Now, an important task is to elucidate how these herbal compounds perturb these pathways by computationally modeling their interactions with their target proteins [46]. Here, we show that unbiased target profiles can be systematically predicted through integrating chemical similarity information and the relatedness of drug targets in the protein-protein interaction network. The target profiles identified by drugCIPHER [29] can provide a comprehensive understanding of the pharmacology effects and side effects or toxicities for a given herbal compound with rigid chemical structure. Computational scoring of target proteins using our approach led to the identification of vitexicarpin-interacting proteins at a genome-wide level. The method is cost effective, highly flexible, and applicable to all kinds of herbal compounds with known chemical structure, and it can be used with mechanisms analysis of any herbal compounds or TCM formulae of interest.

The top 10% target-related proteins of vitexicarpin are significantly enriched in the 38 KEGG pathways. 14 cancer-related pathways and T cell receptor signaling pathway exactly account for the known biological functions of vitexicarpin. Interestingly, VEGF signaling pathway can partly elaborate the Antiangiogenic mechanisms of vitexicarpin, consistent with our experimental results. Of the remaining pathways, Neurotrophin signaling pathway, Insulin signaling pathway, and Type II diabetes mellitus are enriched by target-related proteins of vitexicarpin, suggesting that it may be potentially used for the diabetes therapy. Thus, vitexicarpin's result provides an example of how “target profiles” predicted by drugCIPHER can comprehensively shed light on the mechanisms of action of herbal compounds. 

The available target profile makes it possible to infer the mechanism of a herbal compound of interest by comparing profiles with existing FDA-approved drugs. As shown in Figure 6(a), drugs such as vitamin E, dasatinib, arsenic trioxide, isoproterenol, sorafenib, and indomethacin have similar target profiles with vitexicarpin and these drugs also show certain Antiangiogenic activities [47–52]. The drugCIPHER method can give the measurement of compound-protein associations, including information on direct protein physical interactions and functional interactions. Recent work has shown that drug's effects can pass either within a protein or across several proteins, to achieve specific interactions (enhance or inhibit) along a network [53]. Therefore, functionally interacting proteins with the compounds may be close to directly interacting proteins in the network. In this study, we show that vitexicarpin can inhibit the phosphorylation of SRC and AKT, which partly elucidate the Antiangiogenic action of vitexicarpin, although the direct targets of vitexicarpin are still uncertain and need further investigation. 

In summary, this work suggests that vitexicarpin is an angiogenesis inhibitor *in vitro* and *in vivo*. We successfully applied drugCIPHER to predict and experimentally validate key proteins SRC and AKT in the target network of vitexicarpin, revealing a previously unreported anti-angiogenesis molecular basis of vitexicarpin on vascular endothelial cell migration, apoptosis, and proliferation. This work also highlights the considerable potential for future herb medicine researches in terms of TCM network pharmacology.
